# Influence of substituting soybean meal with moringa seed cake on feed intake, growth performance, digestibility, blood parameters and economics of fattening crossbred calves

**DOI:** 10.1007/s11250-023-03638-9

**Published:** 2023-05-20

**Authors:** Ola G.A. Hassan, Masouda A. Allak, Gamal M. El-Garhy, Gamal A. Mousa

**Affiliations:** Animal Production Department, Faculty of Agriculture, Fayoum University, Fayoum, 63514 Egypt

**Keywords:** Ruminants, Moringa seed cake, Concentrates, Nutritive values, Metabolites

## Abstract

The aim of this study was to investigate the effects of substitution moringa seed cake (MSC) for soybean meal (SBM) in calves’ rations on blood parameters and growth performance. Thirty-two crossbred calves (232 ± 6.75 kg) were divided into 4 groups (8 animals each). All animals were fed a ration of 30% Egyptian clover + 10% corn silage and 60% concentrate mix (CM). The first group (MSC0%) was fed the CM without any MSC (control), while the second (MSC25%), the third (MSC50%), and the fourth (MSC100%) groups were fed on 25%, 50% and 100% MSC by replacing SBM in CM respectively. Results revealed that MSC50% increased (*P* ≤0.05) most of nutritive values and digestibility compared with the tested groups. Also, MSC50% reduced (*P* ≤0.05) feed conversion of dry matter (DM), total digestible nutrient (TDN) and digestible energy (DE) compared to the tested groups. Also, MSC50% increased the total weight gain and net revenue by 13.50% and 22.75%, respectively, compared to control. While MSC100% lowered the total weight gain and net revenue by -7.67% and -4.20%, respectively, compared to control. Total protein and glucose were increased (*P* ≤0.05) by rations with 25% and 50% MSC compared with MSC (0% and100%). Moreover, adding MSC to animals’ rations at different levels improved most of blood metabolites compared to control. Conclusion, moringa seed cake can be used as an alternative protein source to soybean meal in fattening calves rations at level up to 50% to improve growth performance and net profit without adverse effects.

## Introduction

The livestock industry in Egypt is facing many challenges where food shortage comes in the first place (Khayyal et al. 2015). Providing the animals with the nutritional needs to meet their requirements, and achieve the desired profitability is a national concern. In ruminant’s diets, soybean meal (SBM) is most common plant protein source due to the high protein content that reach 45%. Soybean seeds contain a variety of anti-nutritional factors such as trypsin inhibitors, α-amylase inhibiting factor, lectin, goitrin and saponin (Liener 1994). These factors affect the nutritive value, utilization and digestibility of SBM protein (Herkelman et al. 1992), causing metabolic and digestive diseases of animals (Sun et al. 2005), as well as decreased performance traits (Schulze et al. 1993). Furthermore, the high demand of SBM used in livestock industry led to a global increase in the prices of SBM (El- Sayed 2006). Thus, nutrition experts begin to look for an alternative that is rich in their protein content. Many countries have expressed wide interest in identifying possible feed sources, such as shrubs and trees to be used as ruminant diets. Moringa (*Moringa oleifera*) tree has attracted the attention of researchers as an alternative plant protein source that can be fed to ruminant animals (Meireles et al. 2020).

Moringa (family: Moringaceae) is broadly cultivated and can be found across tropics as an indigenous species (Meireles et al. 2020). The annual yield of pods (fruits) reaches 50-70kg from a single moringa tree with an average yield of 12-13 tons of seeds / hectare (Bridgemohan et al. 2014). Moringa seed cake is produced when the oil is extracted from the moringa seed. Moringa seed cake is high in protein content (25%-60%) that differs according to the methods of extraction (Fuglie 2001; El-Naggar et al. 2017). It is nearly free of saponins, tannins, alkaloids, glucosides, lectins, inhibitors of amylase and trypsin however it does contain glucosinolates (Makkar and Becker 1997). Moringa seeds contain a high level of sulfur-amino acids, minerals (calcium, potassium, zinc, iron, selenium, and copper) and vitamins (A, E and C) and essential fatty acids (Makkar and Becker 1997; Mahaman et al. 2022). Also, MSC has high natural antioxidant content like ascorbic acid and flavonoids that possess the antioxidant activity, which enhances the performance of ruminants (Nadeem et al. 2013; Babiker et al. 2017). Moreover, MSC has bioactive agents like mullein and riseofulvin that have antibacterial and antifungal activity against pathogenic bacteria and fungi, and this enhances the activity of favourable microbes in rumen, as a consequence, improving the efficiency of ruminal fermentation, digestibility, and the performance of ruminants (Babiker et al. 2017; Soltan et al. 2017).

Moringa seed cake had been demonstrated to increase post-rumen protein availability by reducing dietary protein breakdown in an *in vitro* rumen study (Folkard et al. 2001; Makkar et al. 2007). Furthermore, Makkar et al. (2007) and Abdel-Rahman et al. (2019) reported that, MSC at levels of 25% and 50% can be used as an alternative protein source to cottonseed meal in the rations of male goats. Studies on the effect of feeding moringa seed cake on growth performance of ruminants are rare. In addition, the level and type of moringa seed cake utilized varied across recently studies. Thus, the current trial was performed to investigate the effects of total and partial replacement of SBM with MSC on feed intake, growth performance, apparent digestibility, blood parameters and antioxidant activity of crossbred cow calves. In addition, simple economic feasibility study of the tested groups was calculated.

## Materials and methods

### Animal management

The study was conducted in the farm and laboratories of Faculty of Agriculture, Fayoum University. Fayoum Governorate, Egypt. The farm of current study is situated in Damou, (29°17´49N and 30°55´21E), Fayoum, and its weather in the winter is dry with rare rain and daily temperature ranging from of 9–22°C.Thirty-two crossbred cow calves (232 ± 6.75 kg, age: 6 months) were treated for internal and external parasites prior to the experiment by doramectin (Dectomax^®^_,_ Zooteis; 1 mL / 50 kg BW) administered in the neck area by intramuscular injection. The calves were maintained under the same management and environmental conditions. All animals of this study were housed in an open shaded yard surrounded by steel fences and supplied with internal steel barriers to separate between animals. The flooring of calves’ pens were concrete-floors and fitted with locally made feed manager. Each calf in the study was kept in an area of 2.2 ×2.8 m^2^. The light bulbs were used daily to provide lighting of 12 h of light for each calf in the experiment. Approvals to use the animals were obtained from the Institutional Animal Care and Use Committee for Fayoum University (FU-IACUC) protocol No. AEC2105.

### Feeding trial

Moringa seed cake (MSC) was produced by extracting the oil from moringa seed using non solvent mechanical cold-press method (Fils 2000). It was purchased from the Egyptian Scientific Society of Moringa, National Research Centre, Egypt. Thirty-two crossbred calves (Baladi (Egyptian) ×Frisian) were divided into four groups (8 animals each) according to their body weight. All groups of animals in this study were fed a ration of 30% Egyptian clover + 10% corn silage and 60% concentrate mix (CM). The first group (MSC0%) was fed the CM without any MSC (control), while the second (MSC25%), the third (MSC50%), and the fourth (MSC100%) groups were fed on 25%, 50% and 100% MSC by replacing soybean meal (SBM) in CM respectively. The composition of the concentrates and chemical analysis of the tested rations are presented in Tables 1 and 2. The nutrient requirements of calves were determined as mentioned by NRC (2000). Feeding trial lasted for 90 days and animals were acclimatized to the tested rations for 15 days before beginning the feeding trial. For each calf in the trial, concentrate mix was daily offered with corn silage at 8.00 am, while Egyptian clover was daily offered at 4.00 pm, and fresh water was available ad libitum via drinking troughs located on side of the pens.

**Table 1:** Composition of concentrates of the tested rations on DM basis

The tested rations^2^				
Items	MSC0%	MSC25%	MSC50%	MSC100%
Ingredients of CM^1^ (g/kg)				
Yellow corn	550	550	550	550
Wheat bran	225	209	195	165
Soybean meal	200	150	100	0
Moringa seed cake	0	66	130	260
Limestone	15	15	15	15
Premix	5.0	5.0	5.0	5.0
NaCl	5.0	5.0	5.0	5.0

^1^CM: concentrate mix

^2^The substitution (0%, 25%, 50% and 100%) of MSC for SBM was based on the percent of protein in SBM (41.75% CP) and MSC (33.21% CP)

**Table 2:** The chemical composition of feedstuffs and the tested rations on DM basis (g kg ^-^ DM)

Items^1^	OM	CP	EE	NFC	Ash	NDF	ADF
Egyptian clover	827.5	172.9	36.8	230.2	172.5	387.6	211.5
Corn silage	889.9	79.3	48.4	279.2	110.1	483.0	406.2
Moringa seed cake	939.1	332.1	113.9	290.6	60.9	202.5	80.3
Soybean meal	871.0	417.5	28.8	305.71	129.0	119.0	72.0
Yellow corn	930.4	81.5	46.8	718.1	69.6	84.0	22.4
Wheat bran	901.8	137.7	34.9	360.2	98.2	369.0	114.6
MSC0%	870.5	155.4	39.5	419.2	129.5	256.4	135.6
MSC25%	875.3	153.8	46.0	417.1	124.7	258.4	135.4
MSC50%	872.9	154.7	42.8	418.1	127.1	257.3	135.5
MSC100%	880.0	152.1	52.5	415.0	120.0	260.4	135.3

^1^OM: organic matter, CP, crude protein, EE: ether extract, NFC: non-fibrous carbohydrates = (100 - (%NDF+ % CP + %EE +%ash ) according to NRC (2001), NDF: neutral detergent fiber and ADF: acid detergent fiber

MSC0%: control ration containing 30% Egyptian clover + 10% corn silage + 60% CM without any MSC; MSC25%: control ration but MSC was substituted for SBM by 25% in CM; MSC50%: control ration but MSC was substituted for SBM by 50% in CM; and MSC100%: control ration but MSC was substituted for SBM by 100 % in CM

### Growth variables and digestibility trial

The tested animals were weighed every 15 days in the morning prior feeding. The total weight gain (the difference between the final and initial weights) and feed conversions were also calculated. Moreover, the nutritive values were calculated according to equations of NRC (2001). In contrast, digestibility trial was performed at the end of the growth trial. Amount of feed consumption was recorded daily, and the dry matter intake (DMI) for each calf was calculated. Feces samples were daily collected from the rectum of each animal for 7 days at the end of the trial, dried overnight at 65 °C in hot air oven, and stored for chemical analysis. Feeds and feces samples were analyzed as described by AOAC (2012) for dry matter (method 930.15), ether extract (method 920.39), organic matter as calculated by ash (method 942.05), and N content (kjeldahl method 955.04). Factor 6.25 was used for crude protein conversion from N values. While, neutral detergent fiber (NDF) and acid detergent fiber (ADF) of feed were determined according to Van Soest et al. (1991) using Fibertect 2010 (Tecator Comp., Sweden). In addition, nutrients digestibility was determined using the technique of acid-insoluble ash (Van Keulen and Young 1977) as the following equation:

$$\textrm{Digestibility}\ \left(\%\right)=100-\left[100\times \frac{\textrm{Silica}\kern0.5em \textrm{in}\ \textrm{feed}}{\ \textrm{Silica}\kern0.5em \textrm{in}\ \textrm{feces}}\times \frac{\textrm{Nutrient}\ \textrm{in}\ \textrm{feces}}{\textrm{Nutrient}\ \textrm{in}\ \textrm{feed}}\right]$$

### Blood sample analysis

Blood samples were collected at weeks 0, 4, 8, and 12 of the trial before morning feeding. Approximately 10 mL of fresh blood from the jugular vein of the calves in all groups. Each blood sample was divided into three tubes: first tube contained EDTA for variables of hematology using a analyzer of hematological parameters (HA-VET CLINDIAG, China) to determine levels of hemoglobin (Hb), red blood cell count (RBCs), white blood cell count (WBCs), lymphocyte count, platelet count (PLT) and mean corpuscular volume (MCV), while the second tube contained potassium oxalate and sodium fluoride for analyzing glucose (Glu).However the third tube was used for serum separation. Clear blood serum was divided into aliquots across 3 Eppendorf tubes and stored at -20 °C for further assays of total protein (TP), albumin (Alb), creatinine, urea, alanine aminotransferase (ALT), aspartate aminotransferase (AST), total lipids (TLs), triglycerides (TG), total cholesterol (CHO), low-density lipoprotein (LDL) and high-density lipoprotein (HDL) using kits (Spectrum Biotechnology, Egypt) and spectrophotometer (T80 UV/VIS PG instrument Ltd., UK).In contrast, total globulin (Glb) was calculated by difference between the total protein and albumin values. Serum total triiodothyronine (T_3_) and total thyroxine (T_4_) were analyzed using the radioimmunoassay technique kit (RIA source Immunoassay S.A., Belgium), and method sensitivities were 0.2 and 9.86 nmol/l for T_3_ and T_4_, respectively. Capacity of serum antioxidant activity was determined using 1, 1-diphenyl-2-picrylhydrazyl (DPPH) (Himedia Laboratories Pvt., Ltd., India) reduction assay (Blois 1958) by adding 20 μl of serum + 10 mM buffer of sodium phosphate (pH 7.4) total volume of 400 μl to 400 μl of 0.1 mM methanol solution of DPPH. At temperature (21 °C) after 30 minutes of incubation, samples absorbance (Abs _sample_) at 520 nm was determined and compared to that of control sample which contains only phosphate buffer and DPPH solution (Abs _control_) and blank sample which contains only serum and phosphate buffer (Abs _blank_). Antioxidant activity (AA %) was estimated using this equation: AA%=100 - {(Abs _sample_ – Abs _blank_ ×100)/Abs _control_} (Garcia 2012).

### Simple economic evaluation

The cost of 1 ton of corn silage (40% DM) and Egyptian clover (14%DM) in all the tested rations was 350 and 1000 Egyptian pound (EGP) respectively. While the cost of 1 ton of CM was 6397.70, 6151.75, 5903.92, and 5416.93 EGP for rations of MSC (0%, 25%, 50% and 100%) respectively (on DM basis).

### Statistical analyses

A one-way analysis of variance followed by Duncan’s multiple range test to measure data once at a time. The model was: Yij = μ + Ti + eij, where Yij is the parameter under analysis, μ is the overall mean, Ti is the influence of treatment (tested rations), and eij is the error of the experiment. While the repeated measurements (the values of hematobiochemical variables) were subjected to using a two-way analysis of variance in a randomized complete block design to consider the influence of the tested rations, time of collection of blood samples, and the interaction between TIME and the tested rations. The analysis was done using the following model: Yij = μ + Ti + Pj + TPij + eij, where Yij is the dependent variable in the study, μ is the overall mean, Ti is the impact of treatments (i = 1, 2, 3, 4), Pj is the TIME effect of collection of blood samples (j = 1, 2, 3, 4), TPij is the interaction between TIME and tested rations, and eij is the experimental error. All data of this study were statistically analyzed using IBM SPSS version 22. All values of this study were showed as mean and standard error (mean ± SE).

## Results

### Digestibility and nutritive values (TDN, SV, DCP and DE) of the tested rations

Digestibility and nutritive values of the tested rations are shown in Table 3. Except EE digestibility, there were significant changes in nutrient digestibility among the tested rations. In particular, MSC50% significantly increased OM, CP, NFC and NDF digestibility compared with the other tested rations. However, ration of MSC100% was recorded the least values in digestibility of OM, EE, NFC and NDF compared with the tested rations. Also, there were insignificant changes in nutrient digestibility and nutritive values in rations of MSC (0%, 25% and 100%). In contrast, the nutritive values of TDN, SV, and DCP (g/kg) were higher (*P* ≤0.05) in MSC50% compared with the other rations of MSC (0%, 25%, and 100%). Digestible crude protein was 110.5, 112.9, 122.7, and 110.0 (g/kg) in rations of MSC (0%, 25%, 50%, and 100%), respectively.

**Table 3:** Digestibility coefficients and nutritive values of the tested rations (DM basis)

Items^1^	The tested rations				± SEM	*p*-value
	MSC0%	MSC25%	MSC50%	MSC100%		
Digestibility coefficients (g/kg)						
OM	699.7^b^	702.3^b^	769.2^a^	690.7^b^	0.35	0.008
CP	710.9^b^	734.3^b^	793.4^a^	723.4^b^	0.19	0.007
EE	833.9	839.4	850.0	824.1	0.24	0.098
NDF	614.7^ab^	620.0^ab^	689.3^a^	603.7^b^	0.73	0.003
NFC	687.4^b^	675.1^b^	741.6^a^	668.0^b^	0.55	0.002
Nutritive values^2^						
TDN(g/kg)	651.7^b^	660.6^b^	711.4^a^	660.9^b^	0.41	<0.001
SV(g/kg)	625.3^b^	630.4^b^	681.0^a^	628.3^b^	0.65	<0.001
DCP(g/kg)	110.5^b^	112.9^b^	122.7^a^	110.0^b^	0.13	<0.002
DE, MCal/kg	2.85	2.89	3.12	2.89	0.11	<0.081

^a-b^: Mean values in the same row with different superscripts are significantly different (*P* ≤0.05)

^1^ *OM* organic matter, *CP* crude protein, *NFC* non-fibrous carbohydrates, *NFE* nitrogen-free extract: total digestible nutrients, *SV* starch value, *DCP* digestible crude protein, *DE* digestible energy, and *SEM* standard error of the mean (*N* = 8)

^2^ Nutritive values were calculated according to equations of NRC (2001)

### Feed conversion and growth performance of fattening calves

No significant changes were observed in daily feed intake of DM, SV, TDN, DE and DCP. However, MSC100% recorded the least value in feed intake compared with the tested rations (Table 4). In addition, MSC50% decreased significantly feed conversions of DM, TDN and DE compared with the tested rations. Furthermore, no significant differences in SV and DCP conversions were observed among the tested groups. In contrast, the average daily gain and total body gain were increased (*P* ≤0.05) in animals of MSC50% ration compared with the tested rations. Also, MSC50% increased the total weight gain by 13.50% compared to control. While MSC100% lowered (*P* ≤0.05) the total weight gain by -7.67% compared to control.

**Table 4:** Influence of moringa seed cake on the growth performance of crossbred calves

Items	The tested rations				± SEM^1^	*P*-value
	MSC 0%	MSC 25%	MSC 50%	MSC 100%		
Average initial weight, kg	232.00	233.75	238.75	235.00	5.55	0.980
Average final weight, kg	336.25^b^	337.50^b^	357.50^a^	331.25^c^	3.70	0.023
Total gain, kg	104.25^b^	103.75^b^	118.75^a^	96.25^c^	2.34	0.031
Daily gain, kg	1.16^b^	1.15^b^	1.32^a^	1.07^c^	0.04	0.012
Average daily feed intake						
Dry matter (kg/head)	6.82	6.90	7.02	6.70	0.06	0.153
Total digestible nutrients (kg/head)	4.53	4.60	4.99	4.43	0.05	0.241
Starch value (kg/head)	4.26	4.35	4.78	4.20	0.05	0.186
Digestible crude protein (g/head)	753.61	779.01	861.35	737.00	9.30	0.064
Digestible energy (Mcal/day)	19.78	19.94	21.90	19.36	0.21	0.250
Feed conversion						
Dry matter (kg feed/kg gain)	5.89^b^	5.99^b^	5.32^c^	6.27^a^	0.20	0.034
Total digestible nutrients (kg feed/kg gain)	3.91^b^	3.98^b^	3.79^c^	4.14^a^	0.12	0.015
Starch value (kg feed/kg gain)	3.68	3.77	3.62	3.94	0.11	0.267
Digestible crude protein (g feed/g gain)	0.65	0.68	0.65	0.69	0.02	0.190
Digestible energy (Mcal feed/kg gain)	17.08^b^	17.30^b^	16.60^c^	18.11^a^	0.13	0.035

^a-c^: Mean values in the same row with different superscripts are significantly different (*P* ≤0.05)

^1^ SEM: standard error of the mean (*N* = 8)

### Blood serum parameters

#### Hematological parameters

**Fig. 1:**
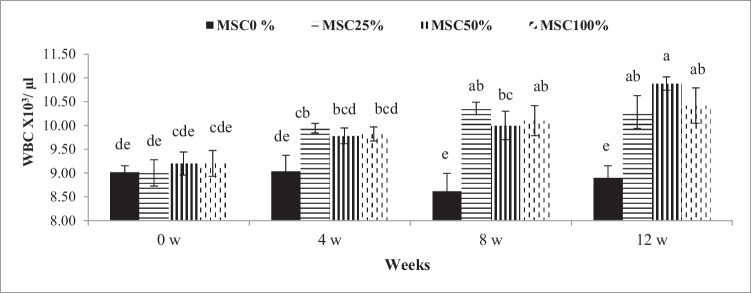
Influence of treatment (levels of moringa seed cake in the tested rations) by time interaction (weeks 0, 4, 8, and 12) on white blood cells (WBCs) of crossbred calves. ^a-e^: Mean values with different superscripts are significantly different (*P* ≤0.05)

Table 5 presented the influences of SBM replacing with MSC on some blood hematological variables in calves. The measured hematological values were different (*P* ≤0.05) at various rations. In particular, Hb, RBCs, WBCs, lymphocytes, and PLT were higher in the rations with MSC compared with those in the control ration. Also, white blood cell count was significantly higher at the 8th and 12th weeks compared with those at the 4th and 0th weeks (Fig. 1). However, MCV was not significantly different between all rations.

**Table 5:** Influence of moringa seed cake on the hematological parameters of crossbred calves

Items^1^	The tested rations				±SEM	Normal range^2^	*p*-value		
	MSC 0%	MSC 25%	MSC 50%	MSC 100%			R	T	R× T
Hb (g/dl)	8.95^b^	9.97^a^	9.99^a^	9.44^ab^	0.22	5–10.5	<0.001	0.026	0.884
RBCs (X106/ mm3)	5.49^b^	6.00^a^	6.01^a^	5.93^a^	0.14	5–10	0.012	0.03	0.085
MCV (fl)	49.89	50.12	50.04	48.19	1.51	40–60	0.782	0.787	0.576
WBC(X103/μl)	8.90^b^	9.89^a^	9.97^a^	9.89^a^	0.22	4–12	<0.001	<0.001	0.025
Lymphocytes (%)	60.00^b^	67.15^a^	66.95^a^	65.40^a^	0.89	40–70	<0.001	<0.001	0.803
Platelet (X103 / μl)	284.70^b^	323.65^a^	287.20^b^	308.65^ab^	10.95	300–800	0.030	0.227	0.109

^a-b^: Mean values in the same row with different superscripts are significantly different (*P* ≤0.05)

^1^Hb: hemoglobin, RBCs: red blood cells, MCV: mean corpuscular volume, WBCs: white blood cells, R: rations, T: time, R× T: interaction between rations and time, and SEM: standard error of the mean (*N* = 8)

^2^The normal range of hematological parameters according to Radostits (2000), Radostits et al. (2007), and the Research Animal Resource (2009)

#### Protein profile and glucose

Blood serum TP, Alb, and Glu concentrations were increased (*P* ≤0.05) by rations with 25% and 50% MSC compared with rations of MSC (0 % and 100%). However, no significant changes in Glb concentration were found among all rations (Table 6).

**Table 6:** Influence of moringa seed cake on the protein profile and glucose of crossbred calves

Items^1^	The tested rations				±SEM	Normal range^2^	*p*-value		
	MSC 0%	MSC 25%	MSC 50%	MSC 100%			R	T	R× T
TP (g/dl)	7.09^b^	7.54^a^	7.57^a^	7.12^b^	0.13	5.7–8.1	<0.001	<0.001	0.594
Alb (g/dl)	3.12^b^	3.54^a^	3.54^a^	3.23^b^	0.07	2.1–3.6	<0.001	<0.001	0.734
Glb (g/dl)	3.97	4.00	4.03	3.89	0.11	2.9–4.9	0.613	0.001	0.539
Glu(mg/dl)	64.78^b^	71.45^a^	71.35^a^	63.94^b^	1.08	45.75	<0.001	0.006	0.320

^a-b^: Mean values in the same row with different superscripts are significantly different (*P* ≤0.05)

^1^TP: total protein, Alb: albumin, Glb: total globulin, Glu: glucose, R: rations, T: time, R× T: interaction between rations and time, and SEM: standard error of the mean (*N* = 8)

^2^The normal range of protein profile and glucose according to Radostits (2000), The Merck Veterinary Manual (2005), the Research Animal Resource (2009), and El-Masry et al. (2018)

#### Lipid profile

**Fig. 2:**
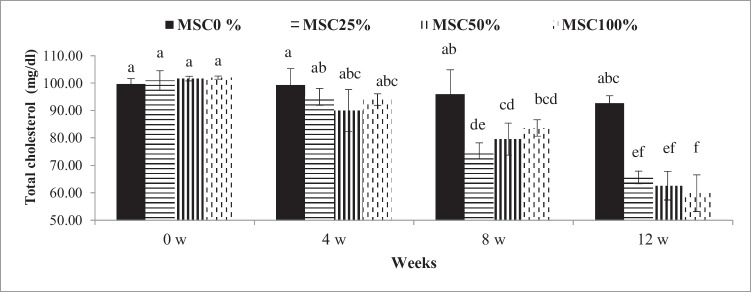
Influence of treatment (levels of moringa seed cake in the tested rations) by time interaction (weeks 0, 4, 8, and 12) on total cholesterol of crossbred calves. ^a-f^: Mean values with different superscripts are significantly different (*P* ≤0.05).

**Fig. 3:**
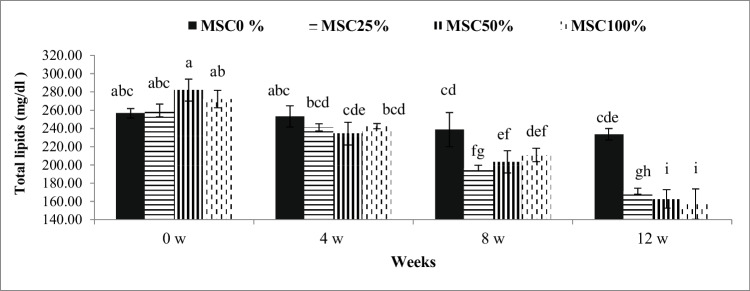
Influence of treatment (levels of moringa seed cake in the tested rations) by time interaction (weeks 0, 4, 8, and 12) on total lipids of crossbred calves. ^a-i^: Mean values with different superscripts are significantly different (*P* ≤0.05)

Table 7 shows that CHO, HDL, LDL, and TL concentrations were significantly improved (*P* ≤0.05) by rations with different levels of MSC (25%, 50%, and100%) compared with control. However, triglycerides were insignificant lower in rations of MSC (25% 50%, and 100%) compared with control. A significant decrease (*P* ≤ 0.05) in CHO and TL concentrations was found at the 8th and 12th weeks compared with those at the 0th and 4th weeks (Figs. 2, 3).

**Table 7:** Influence of moringa seed cake on the lipid profile of crossbred calves

Items^1^	The tested rations				±SEM	Normal range^2^	*p*-value		
	MSC 0%	MSC 25%	MSC 50%	MSC 100%			R	T	R× T
TG (mg/dl)	51.71	48.66	48.42	48.21	2.48	Up to 54	0.325	<0.001	0.600
CHO (mg/dl)	96.92^a^	84.25^b^	83.50 ^b^	84.92^b^	4.36	66–200	<0.001	<0.001	0.027
HDL (mg/dl)	44.08 ^b^	51.85 ^a^	52.33^a^	52.08^a^	1.44	Up to 94	<0.000	<0.001	0.204
LDL (mg/dl)	42.49^a^	22.93^b^	26.63^b^	25.69^b^	4.90	Up to 45	<0.001	<0.001	0.108
TL (mg/dl)	245.54^a^	217.17^b^	220.57^b^	220.54^b^	11.52	-	<0.001	<0.001	0.003

^a-b^: Mean values in the same row with different superscripts are significantly different (*P* ≤0.05)

^1^TG: triglycerides, CHO: total cholesterol, HDL: high-density lipoprotein, LDL: low-density lipoprotein, TL, total lipids, R: rations, T: time, R× T: interaction between rations and time, and SEM: standard error of the mean (*N* = 8)

^2^The normal range of the lipid profile according to Radostits et al. (2007) and Research Animal Resource (2009)

#### Blood serum hormones, kidney function, and liver enzymes

Adding different levels of MSC (25% 50%, and 100%) in calves ration significantly increased concentration of blood serum T_3_ compared with control (Table 8). However, no significant increase in T_4_ concentration was induced by rations of MSC (25%, 50%, and 100 %) compared with control. Moreover, rations of MSC (25% and 50%) reduced (*P* ≤0.05) concentrations of serum urea compared to MSC (0 % and 100%). Furthermore, no significant reduction in creatinine, AST and ALT concentrations was observed in various rations (Table 8).

**Table 8:** Influence of moringa seed cake on the physiological responses of crossbred calves

Items^1^	The tested rations				±SEM	Normal range^2^	*p*-value		
	MSC 0%	MSC 25%	MSC 50%	MSC 100%			R	T	R× T
T_3_ (ng/dl)	162.29^b^	174.86^a^	175.86^a^	174.18^a^	3.50	59–443	<0.001	<0.001	0.144
T_4_ (μg/dl)	8.02	8.58	8.47	8.62	0.30	4.2–8.6	0.367	0.178	0.086
Urea(mg/dl)	19.33^a^	17.08^b^	17.00^b^	18.74^a^	0.73	12.8–57.8	0.019	<0.001	0.110
Creat. (mg/dl)	1.32	1.24	1.20	1.23	0.03	1–2	0.112	0.832	0.159
ALT (IU/L)	20.17	19.08	19.17	19.08	1.17	11–40	0.863	0.380	0.079
AST(IU/L)	87.42	84.25	83.25	86.67	0.94	78–132	0.191	0.015	0.204

^a-b^: Mean values in the same row with different superscripts are significantly different (*P* ≤0.05)

^1^*T*_3_ total triiodothyronine, *T*_4_ total thyroxine, *Creat* creatinine, *ALT* alanine aminotransferase, *AST* aspartate aminotransferase, *R* rations, *T* time, *R*× *T* interaction between rations and time, and *SEM* standard error of the mean (*N* = 8)

^2^The normal range of the physiological responses according to Radostits et al. (2007), the Research Animal Resource (2009), and El-Masry et al. (2018)

#### Antioxidant activity (AA %)

**Fig. 4:**
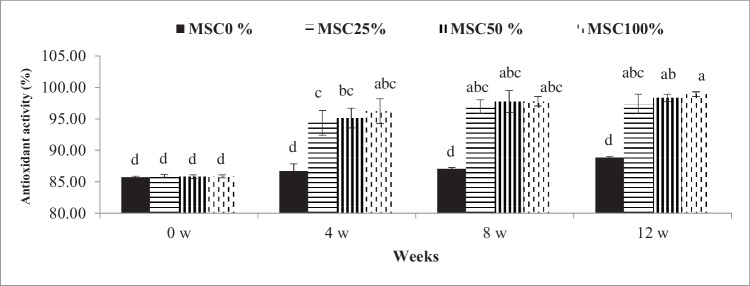
Influence of treatment (levels of moringa seed cake in the tested rations) by time interaction (weeks 0, 4, 8, and 12) on serum antioxidant activity of crossbred calves. ^a-d^: Mean values with different superscripts are significantly different (*P* ≤0.05)

Rations of MSC (25%, 50%, and 100 %) increased (*P* ≤0.05) serum antioxidant activity compared with control throughout the experiment; however, the increases of MSC (25%, 50%, and 100%) were not significant at the 8th and 12th weeks compared with that at the 0th and 4th weeks (Fig. 4).

#### Simple economical evaluation

Adding MSC to the tested rations was reduced the cost of feed consumed (Table 9). The costs of feed consumed/head/period were 2479.75, 2421.90, 2369.25 and 2086.38 EGP for rations of MSC (0%, 25%, 50%, and 100%), respectively. In contrast, the best net revenue (EGP/head/90 day) was EGP 5913.85 with ration of MSC50%, followed by rations of MSC25%, MSC0% and MSC100% with which values were EGP 4840.60, 4817.75 and 4615.12, receptively (Table 9). Both the MSC25% and MSC50% rations increased the net revenue by 0.5% and 22.76%, respectively compared to control. While a high level of moringa seed cake in the ration of MSC100% lowered the net revenue by - 4.20% compared to control.

**Table 9:** Simple economical evaluations of moringa seed cake in crossbred calves’ rations

Items	The tested rations			
	MSC0%	MSC25%	MSC50%	MSC100%
Total weight gain (kg/head/90 day)	104.25	103.75	118.33	96.25
Dry matter consumed (kg/head/90 day)	613.80	621.00	631.80	603.00
Price of one kg DM of the ration (EGP)	4.04	3.90	3.75	3.46
Cost of feed consumed (kg/head/90 day)	2479.75	2421.90	2369.25	2086.38
Total revenue (EGP)^1^	7297.50	7262.50	8283.10	6737.50
Net revenue (EGP)^2^	4817.75	4840.60	5913.85	4615.12

^1^Total revenue, EGP (Egyptian pound) = total weight gain (kg/head/90 day) x EGP 70 (the price of the 1 kg live body weight). ^2^Net revenue (EGP /head/90 day) = total revenue (EGP /head/90 day) - cost of feed consumed (EGP /head/90 day)

## Discussion

In calves fattening systems, one of the major ways to increase farmer’s profit is to decrease the cost of feed intake or enhance feed efficiency. Thus, the objective can be achieved by enhancing feed conversion and weight gains (Mousa et al. 2022). One strategy could be by substituting expensive feeds with alternative sources. In this sense, in the present study SBM was replaced by MSC, which is normally less expensive than SBM in Egypt.

In the current study, the improvement in nutrient digestibility of MSC50% could be attributed to the antimicrobial impact of MSC proteins on fuses the inner and outer membranes bacteria and reducing protein breakdown in the rumen and increase the post-rumen protein supply (Makkar et al. 2007; Ebeid et al. 2022). Also, the improvement in nutrient digestibility may be due to moringa seed cake which is nearly free of saponins, tannins, glucosides, lectins, inhibitors of amylase and trypsin however it does contain glucosinolates (Makkar and Becker 1997). Moringa seed cake also has bioactive agents like mullein and riseofulvin that enhances the activity of favourable microbes in rumen, as a consequence, improving ruminal fermentation, digestibility, and the performance of ruminants (Babiker et al. 2017; Soltan et al. 2017).

Regarding, the low digestibility coefficients of OM, NFC, EE, and NDF in calves fed on MSC100% may be because of the bioactive moieties (cationic proteins) of MSC that elicits an antimicrobial response stronger than necessary and thus inhibit fermentation of rumen (Makkar et al. 2007; Ebeid et al. 2022). In addition, the other reason of low digestibility coefficients in high concentration of moringa seed cake (MSC100%) can also be attributed to the bitter taste caused by glucosinolates (Ebeid et al. 2022; Mahaman et al. 2022). In contrast, the calculated nutritive values (TDN, SV, and DE) are a reflection of increasing nutrient digestibility in all the tested rations. The increasing of DCP in MSC (25% and 50%) may be due to essential amino acids and quality and quantity of moringa proteins (Abdel-Rahman et al. 2019). The obtained nutritive values and digestibility in the study are supported by the findings of El-Naggar et al. (2017) and Abdel-Rahman et al. (2019) , who reported that feeding animals on diets contained moringa seed cake as substitution for soybean meal or cottonseed meal made significant changes in feeding values and digestibility of nutrients.

The current data of feed intake in line with Abdel-Rahman et al. (2019), who found that levels of MSC (25% and 50%) in male goat’s rations as substitution for cotton seed meal increased feed intake. This finding can be attributed to the increase in nutrient digestibility as amino acids, CP, minerals and vitamins (Abd El-Rahman et al. 2011). Also, Nadeem et al. (2013) and Babiker et al. (2017) reported that moringa seed cake has high natural antioxidant content, which will enhance the performance of ruminants. For all feed conversion values, MSC50% displayed the optimum, whereas MSC100% showed the least values. The decreasing of feed conversion in MSC100% may be due to a high level of MSC that increases the concentration of cationic proteins and negatively affect rumen fermentation (Ben Salem and Makkar 2009). Also, the current work of feed conversion confirmed the findings of Abd El-Rahman et al. (2019) who reported that the rations containing moringa seed cake as a new source of protein recorded the best feed conversion. In this study, the increasing in calves’ body weights in rations of MSC (25%and 50 %) are a reflection of increasing nutrient digestibility and feed intake. Also, the significant decrease in daily and total body weight gain in MSC100% may be related to the decreasing in nutrient digestibility and feed intake.

The increasing in the hematological values in rations of MSC (25%, 50%, and 100 %) perhaps because the moringa contain a lot of vitamins especially vitamin C and minerals in particular iron, which promote the synthesis of Hb and amino acids, especially arginine, thereby improving immunity and lymphocyte proliferation (Faye et al. 2011).In contrast, the results of protein fractions and glucose have been supported by previous studies (Aboamer et al. 2020; Zaher et al. 2020), who found that moringa enhanced serum TP, Alb, Glb, and Glu. The increasing in serum TP concentration in rations of MSC25% and MSC50% may be attributed either to the increase of CP digestibility (Kholif et al. 2016) or the high level of protein, vitamins, minerals, and antioxidant components of moringa that decrease protein oxidation (Mohammed et al. 2017). Meanwhile, the increased serum glucose level is due to the high of organic matter digestibility, energy concentration and propionic acid (Kholif et al. 2016). In addition, a linear relation was observed between entry rate of glucose and metabolic energy intake (Annison et al. 2002). On the other hand, the current lipid profile may be due to the hypolipidemic effect of bioactive phytoconstituents such as alkaloids that mediate hypolipidemic action either by stimulating fecal bile acid excretion (Patil et al. 2010) or regulating the activities of lipolytic enzymes (Pop et al. 2018). β-sitosterol, one of the major cholesterol decreasing components of moringa leaf ethanol extract, could also improve the lipid profile (Pop et al. 2018).

In contrast, hormone T_3_ plays an active role in metabolic rate and energy metabolism. The increased levels of free T_3_ and T_4_ reflect the positive impact of moringa, which can be attributed to its high content of antioxidants, especially vitamin C and bioactive components that increase physiological responses (Babiker et al. 2017; Maizuwo et al. 2017). Moringa seed cake has favorable effect on kidney function parameters by decreasing serum urea and creatinine concentrations. These results are in line with Aboamer et al. (2020) and Zaher et al. (2020), who mentioned that using MSC improves kidney function in ruminants. However, the decrease in urea and creatinine levels can be attributed to the protection of feed proteins from rumen degradation and improvement in protein digestion in the intestine (Aboamer et al. 2020). The current results of liver enzymes (ALT and AST) were supported by Zaher et al. (2020), who pointed out that MSC decreased serum ALT and AST concentrations because of its vitamin, flavonoid, glucosinolate, and phenolic acid contents. These components improve antioxidant and anti-inflammatory influences (Ndukaku et al. 2015) and biological activities (Atta et al. 2018). The values of blood parameters were in normal range. Such findings investigate that, there is no side effect concerning using MSC as a replacement for SBM in calves’ rations.

In this study, the improvement of economic efficiency in rations of MSC (25% and 50%) may be attributed to the increased nutrient digestibility caused by the addition of moringa seed cake. This finding has supported by Abdel-Rahman et al. (2019), who found that levels of MSC (25% and 50%) in male goat’s rations as substitution for cotton seed meal generate better net revenue and relative economic efficiency compared with control. The reduced cost of feed consumed with rations of (25%, 50%, and 100%) MSC was due to the lower price of MSC (5000 EGP/ton) compared with the price of SBM was 9300 EGP/ton.

## Conclusion

This study suggested that moringa seed cake can efficiency be used as an alternative protein source to soybean meal in fattening calves rations at level up to 50% to improve growth performance and net profit without any adverse effects on nutrient digestibility and blood metabolites. More experiments were needed to investigate the impact of moringa seed cake at different levels of substitution on growth performance of large ruminants.

## Data Availability

The date that supports the findings of this study are available from the corresponding author on reasonable request.
